# Annual Acknowledgement of Reviewers

**DOI:** 10.1186/s12920-016-0168-7

**Published:** 2016-02-17

**Authors:** Timothy R. Sands

**Affiliations:** BioMed Central, Floor 6, 236 Gray’s Inn Road, London, WC1X 8HB UK

## Abstract

The editors of BMC Medical Genomics would like to thank all our reviewers who have contributed to the journal in Volume 8 (2015).

**Michael Abend**

Germany

**Tomas Adam**

Czech Republic

**Nicola Ancona**

Italy

**Lauren Aoude**

Australia

**Luiz Araujo**

USA

**Joseph Arron**

USA

**Scott Auerbach**

USA

**Charles Auffray**

France

**Yi Ba**

China

**Yongsheng Bai**

USA

**Khaled Barakat**

Canada

**Debmalya Barh**

India

**Donatella Barisani**

Italy

**Vincenza Barresi**

Italy

**Touati Benoukraf**

Singapore

**Andrew Bergen**

USA

**Laura Bernardini**

Italy

**Denis Bertramd**

France

**Levi Beverly**

USA

**Karen Bieback**

Germany

**Miroslav Blumenberg**

USA

**Niccolò Bolli**

Italy

**Stephen Brown**

USA

**Dieter Bulach**

Australia

**Merve Cakir**

USA

**Mario Cannataro**

Italy

**Hannah Carter**

USA

**Luciano Cascione**

Italy

**Isabella Castiglioni**

Italy

**Xiao Chang**

USA

**Marie-Christine Chartier-Harlin**

France

**Peh Yean Cheah**

Singapore

**Yaosheng Chen**

China

**Xi Chen**

USA

**Yuhong Chen**

China

**Deborah Chiabrando**

Italy

**Balram Chowbay**

Singapore

**Chi-Ming Chu**

Taiwan

**Eric Chuang**

Taiwan

**Joon-Yong Chung**

USA

**Frank Cichocki**

USA

**Michael Clark**

USA

**Diego Clemente**

Spain

**Mark Cookson**

USA

**Laurent Corcos**

France

**James Costello**

USA

**Hélène Côté**

Canada

**Luisa Cutilo**

UK

**Lucette Cysique**

Australia

**Elena Czeizler**

Finland

**William Damsky**

USA

**Manish Datt**

India

**Subhajyoti De**

USA

**Vinicio De Jesus Perez**

USA

**Simone De Jong**

UK

**Michiel Dehoon**

Japan

**Don Delker**

USA

**Graham Dellaire**

Canada

**Bertrand Denis**

France

**Darrell Dinwiddie**

USA

**Jesus Dominguez**

USA

**Todd Druley**

USA

**Shiwei Duan**

China

**Wenrui Duan**

USA

**Adam Dupuy**

USA

**Madeleine Durbeej**

Sweden

**Andrea Edlow**

USA

**Richard Edwards**

Australia

**Josiane Eid**

USA

**Alfonso Eirin**

USA

**Antonio Eleuteri**

UK

**Justine Ellis**

Australia

**Jean-Francois Emile**

France

**Ingrid Falnoga**

Slovenia

**Baojian Fan**

USA

**Jane Ferguson**

USA

**Howard Fox**

USA

**Merete Fredholm**

Denmark

**Sabrina Fritah**

Luxembourg

**Florian Frommlet**

Austria

**Manuela Gariboldi**

Italy

**David Gavin**

USA

**Edward Gelmann**

Colombia

**Shanaz Ghandhi**

USA

**Samit Ghosh**

USA

**Kathleen Gilbert**

USA

**Santhosh Girirajan**

USA

**David Goode**

Australia

**Jan Oxholm Gordeladze**

Norway

**Rita Graze**

USA

**Mickael Guedj**

France

**Camille Guillerey**

Australia

**Chris Gunter**

USA

**Rongjun Guo**

USA

**Shicheng Guo**

USA

**John Halsall**

UK

**Zhi Han**

China

**Quan-Ze He**

China

**Dennie Hebels**

Netherlands

**Robert Hegele**

Canada

**Axel Hillmer**

Singapore

**Lisa Hines**

USA

**David Hodson**

UK

**Anelia Horvath**

USA

**Galen Hostetter**

USA

**Nan Hu**

USA

**Mini Huang**

Singapore

**Adriana Huertas Vazquez**

USA

**Lisa Hui**

Australia

**Geoffrey Hunter**

Canada

**Mark Ibberson**

Switzerland

**Karen-Amanda Irvine**

USA

**Takatsugu Ishimoto**

Japan

**Rodolfo Iuliano**

Italy

**Patrick Jay**

USA

**Jaroslaw Jendrzejewski**

Poland

**Jakob Jensen**

USA

**Taylor Jensen**

USA

**Yun Ji**

USA

**Zhiliang Jia**

USA

**Glen Jickling**

USA

**Mizuho Kalabis**

USA

**Henry Kaminski**

USA

**Peter Kannu**

Canada

**Sara Katsanis**

USA

**Chiea Chuen Khor**

Singapore

**Hoguen Kim**

South Korea

**Sung-Soo Kim**

South Korea

**Makoto Kinoshita**

Japan

**David Kisor**

USA

**Robert Klein**

USA

**Viktor Koelzer**

Switzerland

**Kenichi Kohashi**

Japan

**Takashi Kohno**

Japan

**Konstantinos Krampis**

USA

**Marieke Kuijjer**

USA

**Liang-Chuan Lai**

Taiwan

**Xun Lan**

USA

**Mario Lauria**

Italy

**Rozen Le Panse**

France

**Ordan Lehmann**

Canada

**Zhengdeng Lei**

Singapore

**Jingyi Jessica Li**

USA

**Shibo Li**

USA

**Weng Khong Lim**

Singapore

**Jörg Linde**

Germany

**Michael Linnebacher**

Germany

**Julian Little**

USA

**Hsuan Liu**

Taiwan

**Xiangtao Liu**

USA

**Dazhi Liu**

USA

**Mats Ljungman**

USA

**William Lockwood**

Canada

**Stacie Loftus**

USA

**Marie Loh**

Singapore

**Ruibang Luo**

China

**Jun-Hang Luo**

Canada

**Beverly Lyn-Cook**

USA

**Milind Mahajan**

USA

**Sherif Mahmoud**

Canada

**Simeen Malik**

Singapore

**Veronika Mancikova**

Spain

**Claudia Manzoni**

UK

**Chaymaa Marouf**

Morocco

**Saber Masmoudi**

Tunisia

**Astuji Matsuyama**

Japan

**Tom Mayo**

UK

**Matthew McCall**

USA

**Michael McConnell**

USA

**Pall Melsted**

USA

**Jia Meng**

China

**David Meyre**

Canada

**Eugenia Migliavacca**

Switzerland

**Suzanne Miller**

UK

**Sherri Millis**

USA

**Francesco Moccia**

Italy

**Halima Moncrieffe**

USA

**Sandro Morganella**

Italy

**Deborah Morris-Rosendahl**

UK

**Jarrett Morrow**

USA

**Sara Mostafavi**

Canada

**Neelanjan Mukherjee**

Germany

**Ronny Myhre**

Norway

**Fatiha Nassir**

USA

**Petr Nazarov**

Luxembourg

**Isabel Nepomuceno-Chamorro**

Spain

**Richard Newton**

UK

**Joanne Ngeow**

Singapore

**Kwangsik Nho**

USA

**Shin-Ya Nishio**

Japan

**Christopher Oakes**

USA

**Sofia Oliveira**

Portugal

**Francisco José Ortega**

Spain

**Vanessa Ota**

Brazil

**Vural Ozdemir**

Turkey

**Hyojung Paik**

South Korea

**Shashi Bhushan Pandit**

India

**Philippos Patsalis**

Cyprus

**Magdalena Pawelkowicz**

Poland

**Jacob Peedicayil**

India

**Lucy Peipins**

USA

**Luke Pilling**

UK

**Dietmar Pils**

Austria

**David Pisetsky**

USA

**Piet Pretorius**

South Africa

**Trevor Pugh**

Canada

**Aditi Qamra**

Singapore

**Li Qian**

USA

**Huanlong Qin**

China

**Zhengzhong James Qu**

Singapore

**John Quillin**

USA

**Paula Ramos**

USA

**Linda Reis**

USA

**Stephen Robertson**

New Zealand

**Xiaoyang Ruan**

USA

**Kalle Rytkonen**

USA

**Anna Sablina**

Belgium

**Latha Satish**

USA

**André Scherag**

Germany

**Michael Schubert**

UK

**Claudine Seeliger**

Germany

**Meenu Sharma**

Canada

**Chad Shaw**

USA

**Michael Shtutman**

USA

**Alejandro Sifrim**

UK

**Birgit Sikkema-Raddatz**

Netherlands

**Sher Singh**

Taiwan

**Guramrit Singh**

USA

**Donna Slater**

Canada

**Caren Smith**

USA

**Charlotte Soneson**

Switzerland

**Boryana Stamova**

USA

**Katrina Steiling**

USA

**Adam Stevens**

UK

**Zhifu Sun**

USA

**Jingchun Sun**

USA

**Kevin Sweet**

USA

**Min-Han Tan**

USA

**Iain Tan**

Singapore

**EC Tan**

Singapore

**Daniel Tan**

Singapore

**Nicholas Tan**

USA

**Xing Tang**

USA

**Jie Tang**

USA

**Jie Tang**

USA

**Cenny Taslim**

USA

**Christopher Taylor**

USA

**Ivana Ticha**

Czech Republic

**Maarit Tiirikainen**

USA

**Gabriele Togliatto**

Italy

**Amanda Toland**

USA

**Christi Van Asperen**

Netherlands

**Jeroen Van De Peppel**

Netherlands

**George Vassiliou**

UK

**Henk Verheul**

Netherlands

**Eranga Vithana**

Singapore

**Logan Walker**

New Zealand

**Christopher Walker**

USA

**Tao Wang**

USA

**Zefeng Wang**

USA

**Tao Wang**

USA

**Zhibin Wang**

USA

**David Wang**

USA

**Ito Wasito**

Indonesia

**Tuangsit Wataganara**

Thailand

**Bernhard Weber**

Germany

**Ping Wei**

China

**Richard White**

USA

**Esther Wilk**

Germany

**John Williams**

USA

**Paul Wilson**

USA

**Christopher Wong**

Singapore

**Gregory Wray**

USA

**Yankai Xia**

China

**Michael Xie**

Canada

**Hongqi Xin**

USA

**Chao Xing**

USA

**Zu Ying Xiong**

China

**Bo Yan**

China

**Henry Yang**

Singapore

**Kai Ying**

USA

**Joon-Ho Yu**

USA

**Jinsheng Yu**

USA

**Jinsheng Yu**

USA

**Guocheng Yuan**

USA

**Holm Zaehres**

Germany

**Adam Zawada**

Germany

**Zheng Zha**

USA

**Ming Zhang**

China

**Ping Zhou**

USA

**Haidong Zhu**

USA

**Jing Zhu**

USA

